# Machine learning based tissue analysis reveals Brachyury has a diagnosis value in breast cancer

**DOI:** 10.1042/BSR20203391

**Published:** 2021-04-06

**Authors:** Kaichun Li, Qiaoyun Wang, Yanyan Lu, Xiaorong Pan, Long Liu, Shiyu Cheng, Bingxiang Wu, Zongchang Song, Wei Gao

**Affiliations:** 1 Shanghai Fourth People’s Hospital Affiliated to Tongji University, Shanghai 200434, China; 2 Tianyou Hospital Affiliated to Tongji University, Shanghai 200331, China

**Keywords:** Brachyury, Breast cancer, decision tree, machine learning

## Abstract

Background: The aim of the present study was to confirm the role of Brachyury in breast cancer and to verify whether four types of machine learning models can use Brachyury expression to predict the survival of patients.

Methods: We conducted a retrospective review of the medical records to obtain patient information, and made the patient’s paraffin tissue into tissue chips for staining analysis. We selected 303 patients for research and implemented four machine learning algorithms, including multivariate logistic regression model, decision tree, artificial neural network and random forest, and compared the results of these models with each other. Area under the receiver operating characteristic (ROC) curve (AUC) was used to compare the results.

Results: The chi-square test results of relevant data suggested that the expression of Brachyury protein in cancer tissues was significantly higher than that in paracancerous tissues (*P*=0.0335); patients with breast cancer with high Brachyury expression had a worse overall survival (OS) compared with patients with low Brachyury expression. We also found that Brachyury expression was associated with ER expression (*P*=0.0489). Subsequently, we used four machine learning models to verify the relationship between Brachyury expression and the survival of patients with breast cancer. The results showed that the decision tree model had the best performance (AUC = 0.781).

Conclusions: Brachyury is highly expressed in breast cancer and indicates that patients had a poor prognosis. Compared with conventional statistical methods, decision tree model shows superior performance in predicting the survival status of patients with breast cancer.

## Background

More and more researchers try to apply the machine learning algorithm to the medical field, because the machine learning algorithm can be clearly distinguished from the reliability of results, and work by finding patterns in data obtained from diagnostic tests, which can be used to predict clinical outcomes. For example, machine learning can be used to predict the response of melanoma patients to PD1 antibody treatment [1]. Besides, researchers can use machine learning algorithm to improve the accuracy of medical imaging diagnosis of important diseases [2,3].

Brachyury is a T-box transcription factor, which has the function of driving EMT. Although EMT exists during the normal development of early embryonic cells, EMT in tumor cells is more active. Therefore, EMT makes tumor cells more invasive and resistant. Although in the previous study, we have found that Brachyury can promote the occurrence of EMT of breast cancer cells [4,5], there are no clinical data supporting this. In the present study, we prepared paraffin tissue from 303 cases of breast cancer tissues, constructed tissue chips, and tried to evaluate the value of Brachyury protein expression in breast cancer prognostic analysis using machine learning algorithms.

## Methods

### Clinical samples and immunohistochemistry

From 2002 to 2014, we collected paraffin specimens of cancer and paracancerous tissues of patients with breast cancer from Shanghai Changhai Hospital, Shanghai Ruijin Hospital, Shanghai Xinhua Hospital and Shanghai Huangpu District Central Hospital.

Inclusion criteria: The pathological diagnosis was based on a woman who was confirmed as primary breast cancer by thick needle aspiration biopsy or surgical incision of biopsy tissue samples, and she was not more than 70 years old. Besides, her blood test indexes and cardiopulmonary function were basically normal. Exclusion criteria: clinical stage IV.The study including 573 cases of primary breast cancer tissues and 29 cases of paracancerous normal tissues. Finally, we successfully constructed seven tissue chips, of which six were cancer tissue chips, with a total of 303 cases; one was a paracancerous normal tissue chip, with a total of 29 cases. All cases were diagnosed by comprehensive pathology and definitely confirmed as breast cancer. All patients received systemic local and/or systemic treatment including radiotherapy, surgery, chemotherapy and endocrine therapy. We obtained hospitalization number and pathology number from the medical record room, collected all original medical records corresponding to patients through the hospital internal database, collated the data of breast cancer patients, and classified the statistics according to specified indicators, including clinical characteristics, lymph node metastasis, and TNM staging. We used the streptomycin avidin-peroxidase (HRP) complex method to determine the distribution of antigens in tissues and cells through the biotin streptavidin reaction. The results were judged by double-blind method. Without knowing the patient’s clinical data, two experienced pathologists judged separately and reviewed the inconsistent results. The study was approved by the ethics committee and institutional review board of Shanghai Fourth People’s Hospital Affiliated to Tongji University. The ethics approval number is 2020031001 and all the participants in the study gave written informed consent.

### Scoring criteria for immunohistochemistry

For Brachyury-positive cells, the positive staining was light yellow, brownish-yellow, and brown, which were located in the nucleus. The results of immunohistochemistry were evaluated using a two-level scoring method. According to the degree of staining, positive cells ≤ 5% were judged as 0 points, 6–25% were judged as 1 point, 26–50% were judged as 2 points, and 51–75% were judged as 3 points, and >75% were judged as 4 points. For staining intensity, noncoloring was judged as negative and counted as 0 points, light brown was judged as weak positive (+) and counted as 1 point, dark brown was judged as strong positive (3+) and counted as 3 points, and staining between weak positive and strong positive was judged as (2+) and counted as 2 points. The comprehensive calculation was based on the product of staining intensity and percentage of positive cells, of which 0 points were judged as (-), 1–4 points were judged as (+), 5–8 points were judged as (2+) and 9–12 points were judged as (3+). A total score of 0–4 points was considered negative, and a total score of 5–12 points was considered positive.

### Machine learning methods

#### Decision tree (DT)

Decision tree (DT) is a supervised machine learning algorithm, which is used to create a model for predicting the target variable value based on several input variables by repeated classification [6]. The model consists of node, branch, and leaf, resembling a tree structure. Sorting of each node is accomplished by using a mathematical method called attribute selection. The measurement of attribute selection is very important for accuracy. The criteria used for the selection are information *gain* and *gini* index, which reflect the reduction in entropy due to sorting of the attribute.

#### Artificial neural network (ANN)

An artificial neural network (ANN) is a computing system inspired by animal brains neuron network. Artificial neural network consists of an input layer, one or more hidden layers, and an output layer [7]. There are neurons in each layer, and they connected with neurons in adjacent layers. Every neuron has its own weight in its initial state. Data are transmitted to neurons in the next layer until they reach the output. In a whole recursive process, the weights are corrected to obtain better accuracy. After the learning process, the optimized weights are provided to the artificial neural network.

#### Random forest (RF)

Random forest (RF) is a tree-based machine learning method for classification, regression and other tasks that operated by constructing a multitude of decision trees [8]. It created many subsets by random sampling that is also called bootstrap aggregation.

#### Logistic regression (LR)

Logistic regression (LR), a common statistical method, was used to evaluate the relationship between categorical variables. It is widely applied in evaluating risk factors or predicting likelihoods of diseases in medical research.

### Data analysis

We used the mice package in R to perform multiple imputation on missing data. First, SPSS 21.0 statistical software was used to perform univariate analysis on the data, and *P*<0.05 on both sides indicated that the difference was statistically significant. Then, different statistical methods were used according to the specific conditions of the data. Mann–Whitney *U* nonparametric test was used to analyze the relationship between the expression of Brachyury protein and age; Pearson X^2^ test or Fisher exact probability test was used to analyze the Brachyury expression in cancer tissues and paracancerous tissues; McNemar’s test was used to analyze the Brachyury matched expression in cancer tissues and paracancerous tissues; and *P*<0.05 on both sides indicated that the difference was statistically significant. Subsequently, we calculated the person correlation coefficient between each variable, compared the relationship between each variable and the patient’s prognosis, and then selected the variables suitable for modeling. We used logistic regression, random forest, decision tree, and neural network algorithms to build clinical prediction models. All the above models were implemented using R language.

## Result

### Patient characteristics and immunohistochemical results

**Figure 1:**
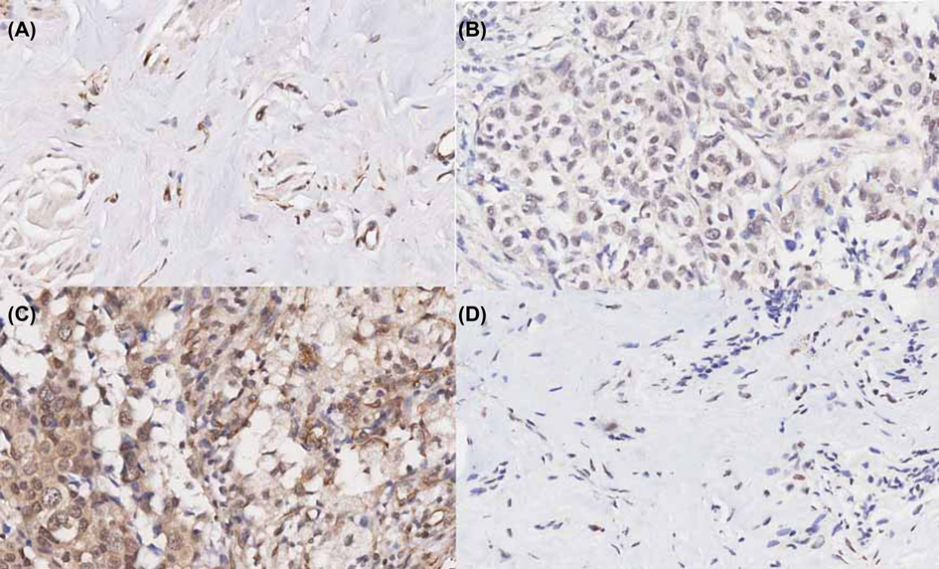
Expression of Brachyury protein in breast cancer tissues and paracancerous tissues (**A**) cancer tissue +, (**B**) cancer tissue ++, (**C**) cancer tissue +++, and (**D**) paracancerous tissue +

**Figure 2:**
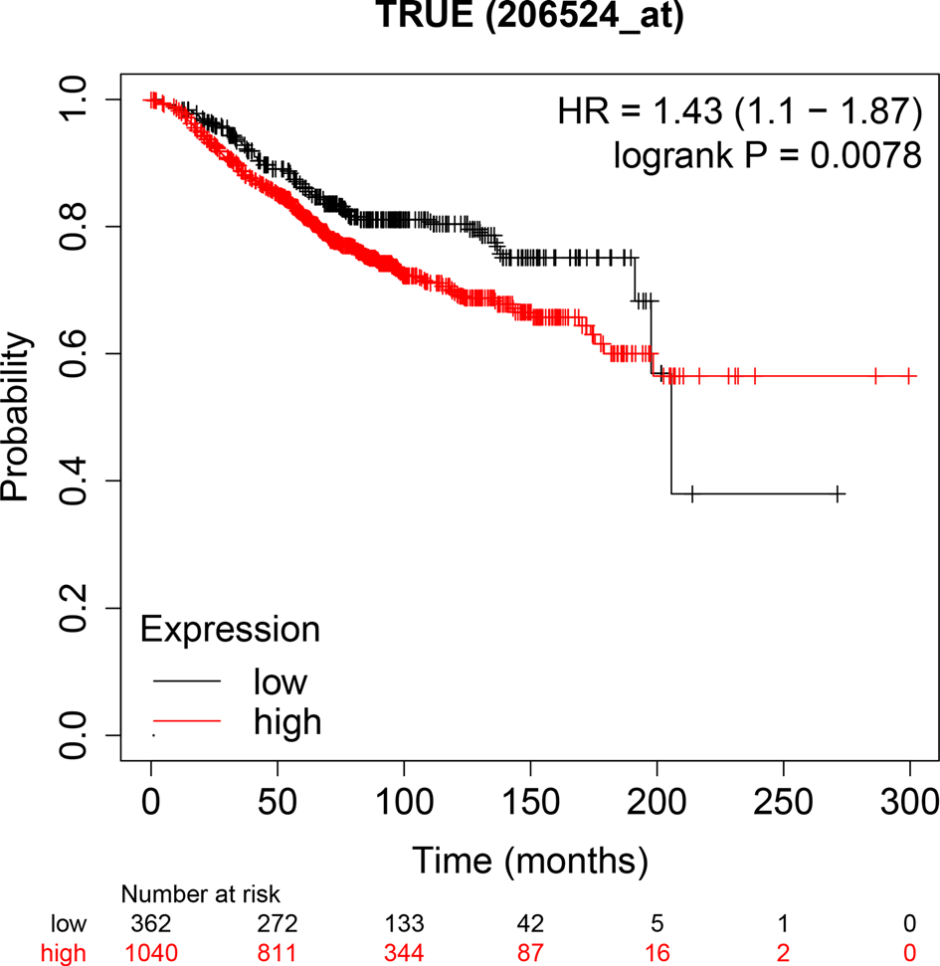
Survival values of Brachyury expression generated by the Kaplan–Meier (KM) plotter

Our final tissue chips contained a total of 332 cases of breast cancer samples, including 303 cases of cancer tissues and 29 cases of paracancerous normal tissues, 28 of which were paired samples. The Brachyury protein expression was detected by IHC assay in breast cancer. Results showed that Brachyury, which was embedded in the nucleus and nuclear envelope, was overexpressed in breast cancer tissues (Supplementary Figure S1). We conducted Pearson X^2^ test on the positive expression of Brachyury in cancer tissues and paracancerous tissues. The results showed that the positive expression of Brachyury in cancer tissues was significantly higher than that in paracancerous tissues (Table 1 and Figure 1). After that, we also conducted McNemar’s test on the paired samples, and the results showed that the difference in the expression of Brachyury protein between cancer tissues and paracancerous tissues in the same breast cancer case was statistically significant (Table 2). Combined with our previous results, this further clarified that Brachyury protein expression might be related to the patient’s prognosis. We also explored the relationship between Brachyury gene expression and patient survival in the KMPLOTTER database. The results showed that patients with high Brachyury expression had a poorer prognosis than patients with low Brachyury expression (Figure 2).

**Table 1:** Expression of Brachyury protein in breast cancer and paracancerous tissues

	*N*	Negative	Positive	X_square	*P*_value
Tumor	303	209(68.98)	94(31.02)	4.5181	0.0335
Paracancerous	29	26(89.66)	3(10.34)		

**Table 2:** Expression of Brachyury protein in paired cases of breast cancer and paracancerous tissues

	Negative	Positive	*N* (%)	X_square	*P*_value
Paracancerous negative	12(42.86)	13(46.43)	25(89.29)	8.6429	0.0033
Paracancerous positive	1(3.57)	2(7.14)	3(10.71)		
*N* (%)	13(46.43)	15(53.57)	28(100)		

### Correlation between brachyury expression and clinical characteristics in breast cancer

The correlation between Brachyury expression and pathological parameters in breast cancer was analyzed. The results suggested that the differences between Brachyury protein expression and different ages, histological grade, tumor size, presence or absence of lymph node metastasis, AJCC stage, pathological diagnosis, and PR expression status could not be considered statistically significant, and the differences between Brachyury protein expression and ER (*P*=0.0392) and HER2 (*P*=0.0572) expressions could be considered statistically significant (Table 3). Survival prognosis is one of the important basis for clinical decision to implement specific interventions for patients with breast cancer, but there is currently no recognized gold standard for prognostic analysis of breast cancer.

**Table 3:** Relationship between Brachyury protein expression and clinical pathological parameters of breast cancer

		Negative	Positive	X^2^	*P*
Age	Median age	53(30–83)	54(30–84)		0.1302
	AJCC stage				
Stage:1	I	43(63.24)	25(36.76)	1.9009	0.3866
Stage:2	II	112(69.14)	50(30.86)		
Stage:3	III	54(73.97)	19(26.03)		
	Histological stage				
Hyphology_class_new_y:1	I	4(66.67)	2(33.33)	0.1568	0.9246
Hyphology_class_new_y:2	II	147(69.67)	64(30.33)		
Hyphology_class_new_y:3	III	58(67.44)	28(32.56)		
	Menstrual status				
Menopause:0	Menopause	111(65.29)	59(34.71)	2.0783	0.1494
Menopause:1	Not menopausal	98(73.68)	35(26.32)		
	Tumor size				
Tumor_max_diameter: ≤2 cm	≤2 cm	75(65.79)	39(34.21)	3.2908	0.1929
Tumor_max_diameter: 2.1–5 cm	2.1–5 cm	116(69.05)	52(30.95)		
Tumor_max_diameter: >5 cm	>5 cm	18(85.71)	3(14.29)		
	Lymph node metastasis				
Lymph_node: 0	0	118(69.41)	52(30.59)		
Lymph_node: 1–3	1–3	45(67.16)	22(32.84)		
Lymph_node: 4–9	4–9	27(77.14)	8(22.86)		
Lymph_node: ≥10	≥10	19(61.29)	12(38.71)	2.0645	0.5591
	Molecular type				
Molecular.type: 1	Luminal A	48(60.76)	31(39.24)	8.1353	0.0433
Molecular.type: 2	Luminal B	7(58.33)	5(41.67)		
Molecular.type: 3	Her2 overexpression	31(86.11)	5(13.89)		
Molecular.type: 4	Triple negative	123(69.89)	53(30.11)		
	ER				
ER_value_new_y: 1	-	154(72.64)	58(27.36)	3.8781	0.0489
ER_value_new_y: 2	+	55(60.44)	36(39.56)		
	PR				
PR_value_new_y: 1	-	184(70.5)	77(29.5)	1.5556	0.2123
PR_value_new_y: 2	+	25(59.52)	17(40.48)		
	Her2				
HER2: -	-	137(65.55)	72(34.45)	3.1985	0.0737
HER2: +	+	72(76.6)	22(23.4)		

**Figure 3:**
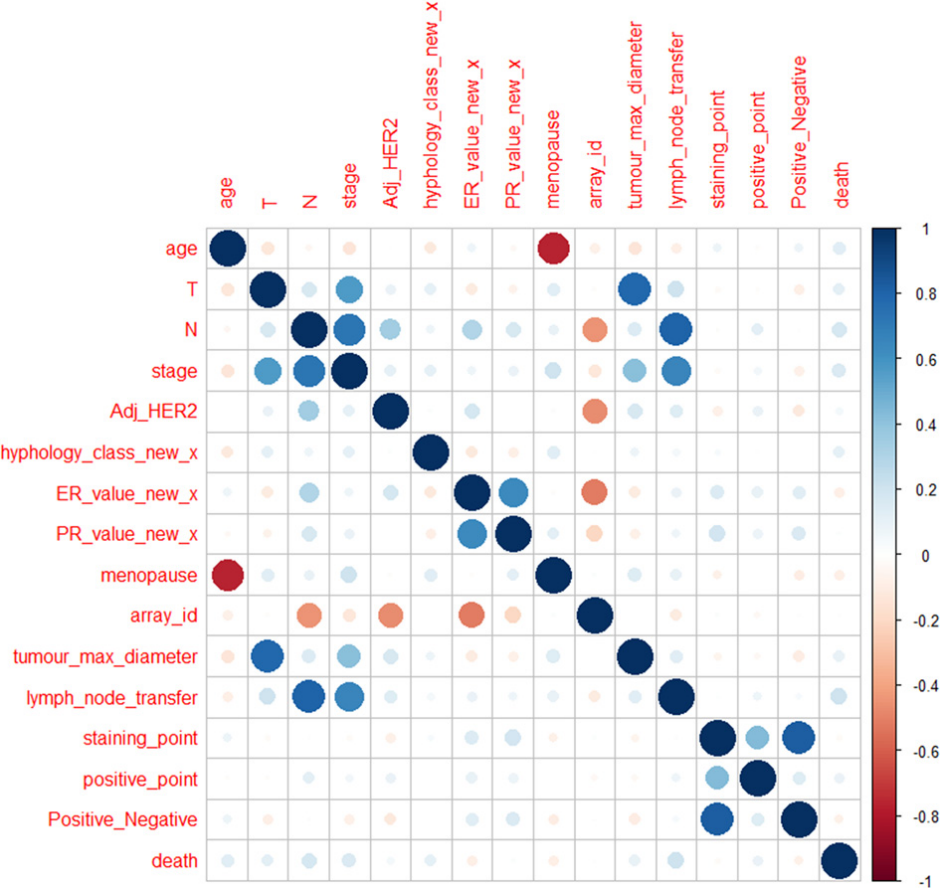
Pearson correlation matrix of data of patient with breast cancer

We used the Pearson correlation coefficient to test the correlation between various variables in patients with breast cancer. The results showed that even the common pathological staging of breast cancer that frequently used in clinical practice, such as molecular typing or TNM staging, had little correlation with the survival rate of patients (Figure 3).

### The performance of machine learning models

**Figure 4:**
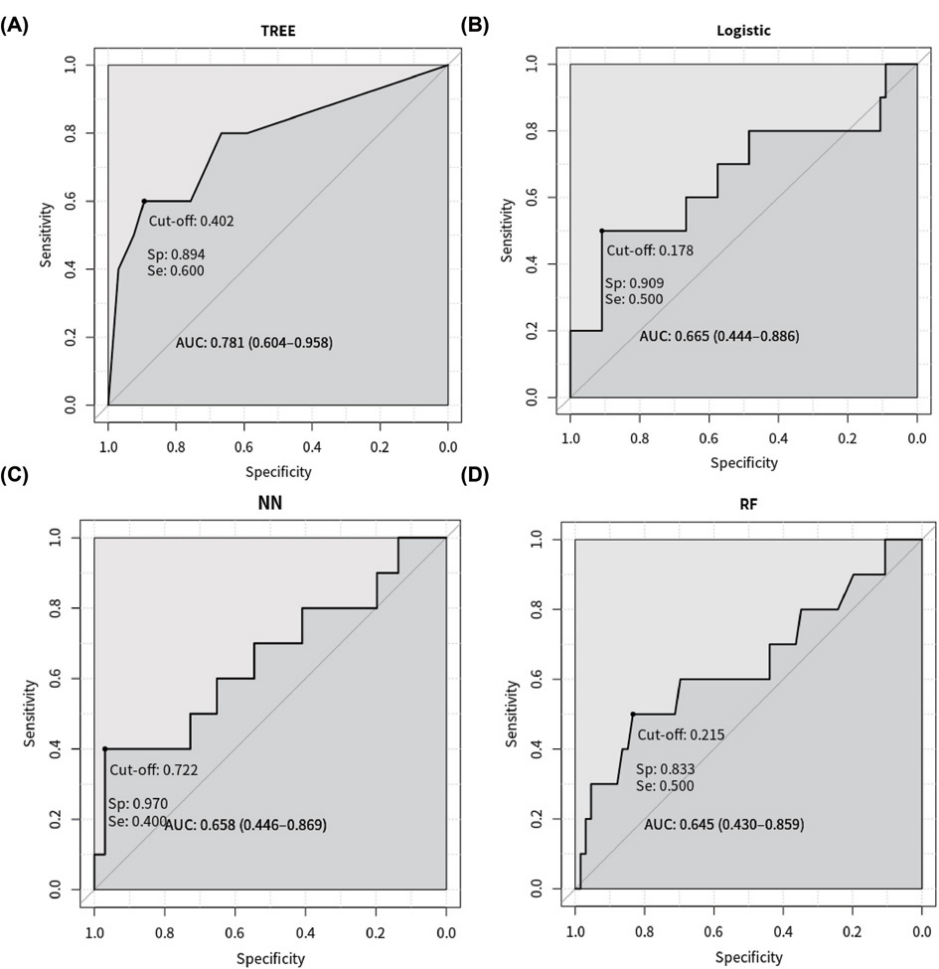
ROC curves used to assess model performance (**A**) Decision tree (**B**) Logistic regression (**C**) Neural network (**D**) Random forest

We used 75% (227 cases) of samples as the training set, and 25% (75 cases) of samples as the test set, and employed machine learning algorithms random forest, decision tree, neural network and logistic regression, all of which were superior to algorithms of conventional statistical methods, to consider Brachyury expression and other clinical variables as predictors to construct clinical predictive models for prognostic analysis of breast cancer. The results showed that the decision tree model performed best, with AUC = 0.781, sensitivity = 0.6, and specificity = 0.894 (Figure 4A), while the other three models had AUCs less than 0.7, of which logistic regression AUC = 0.665, sensitivity = 0.5, and specificity = 0.909 (Figure 4B); neural network AUC = 0.658, sensitivity = 0.4, and specificity = 0.970 (Figure 4C); random forest AUC = 0.645, sensitivity = 0.5, and specificity = 0.833 (Figure 4D). The ROC curve of decision tree model showed the highest accuracy, which indicated that it was feasible and effective to integrate the clinical variables of the patients and the pathological detection results of Brachyury as a comprehensive model for predicting the survival of patients with breast cancer.

## Discussion

Brachyury is one of the members of the T-box transcription factor family. Our previous study has found that Brachyury in breast cancer cells can act on SIRT1 to promote tamoxifen resistance [9], indicating that Brachyury may be a therapeutic target for breast cancer. In triple negative breast cancer, Brachyury expression is also higher than normal tissues [10]. Brachyury can improve the invasive ability of breast cancer cells [11], block the cell cycle process, and mediate the development of tumor drug resistance [12]. Brachyury down-regulation or knockout can increase the sensitivity of tumors to chemoradiation [13], indicating that Brachyury plays an important role in the development of breast cancer. In the present study, we used Tissue microarray technology to detect 303 postoperative breast cancer tissue samples, and the results showed that the Brachyury expression in breast cancer tissues was higher than that in paracancerous tissues. More interestingly, we found that the Brachyury expression was related to the molecular typing of breast cancer, especially the expression status of ER, which provided clinical data support for our previous point that Brachyury expression could promote patients’ resistance to tamoxifen. This will encourage us to further explore the mechanism by which Brachyury causes tamoxifen resistance and evaluate its potential as a target to reverse tamoxifen resistance.

Studies have shown that single biomarker is often not very accurate in guiding clinical practice. For example, DNA damage repair capacity, tumor microenvironment, and pdl1 expression are often used together to predict PD-1/PD-L1 checkpoint inhibitors in patients. Previous study reported that the expression level of Brachyury combined with status of tumor-infiltrating CD8+ and FOXP3+ lymphocytes is used to predict the therapeutic effect of radiotherapy and chemotherapy [14]. Lee et al. have also found that high Brachyury expression in primary breast cancer can be used as a poor prognostic factor for breast cancer [15]. In the present study, we considered immunohistochemical staining scores of Brachyury together with the prognostic analysis indicators commonly used in the clinical practice, such as ER expression and Her2 expression. The results suggested that the results of multiple indicators were better than that of single indicator, which was consistent with the previous report. This also suggests that the combined expression levels of Brachyury and ER expression have the potential to predict more accurately resistance to TAM.

Our study showed that decision tree model was better than conventional multivariate regression statistical models, and also better than other machine learning models. This might be due to the fact that we converted the variables into grading variables as much as possible during the research process. Besides, our model using machine learning to predict disease outcome with comparable sample sizes. Edmond et al. developed a morphological classifier based on machine learning to distinguish different levels of epithelial dysplasia in Barrett’s esophagus [16]. Another study used immunohistochemical results from 131 patients with breast cancer to explore biomarkers of breast cancer and verified them in 65 cases of samples [17]. Shipp et al. also used 77 samples to predict the outcome of patients with diffuse large B-cell lymphoma [18]. Nevertheless, larger sample size might create a more accurate model. The present study including 303 patients with breast cancer predicts survivals of patients with breast cancer with noteworthy performance.

Our results were not intended to indicate that we had obtained a perfect classifier. One of the major disadvantages of the present study was that, although we found that Brachyury expression was related to the molecular typing of breast cancer, our limited sample size was not enough to support our use of machine learning models in different molecular typing of breast cancer to predict the impact of Brachyury staining and other pathological parameters on the survival of patients with breast cancer. In subsequent studies, we plan to further collect samples of ER-positive patients with breast cancer for Brachyury staining to improve our prediction model. In addition, due to the lack of intelligibility of the output of machine learning algorithm, our study does not clarify how Brachyury expression is related to the expression of ER, which also needs to be further explored in future study [19].

## Conclusion

We further clarified the relationship between Brachyury expression and ER in clinical samples. At the same time, we also found that one of the machine learning methods, decision tree, could effectively use Brachyury expression to predict the prognosis of patients with breast cancer, and its accuracy was higher than that of conventional statistical methods.

## Supplementary Material

Supplementary Figure S1

## Data Availability

The datasets generated during and/or analyzed during the present study are available from the corresponding author on reasonable request.

## Abbreviations

AUC: area under the receiver operating characteristic curve
ER: estrogen receptor
HRP: streptomycin avidin-peroxidase
OS: overall survival
ROC: receiver operating characteristic
