# Supplementary figures and images for: Machine learning based tissue analysis reveals Brachyury has a diagnosis value in breast cancer
Source: Biosci Rep. 2021 Apr 6;41(4):BSR20203391. doi: 10.1042/BSR20203391 (PMC8024874; doi:10.1042/BSR20203391)

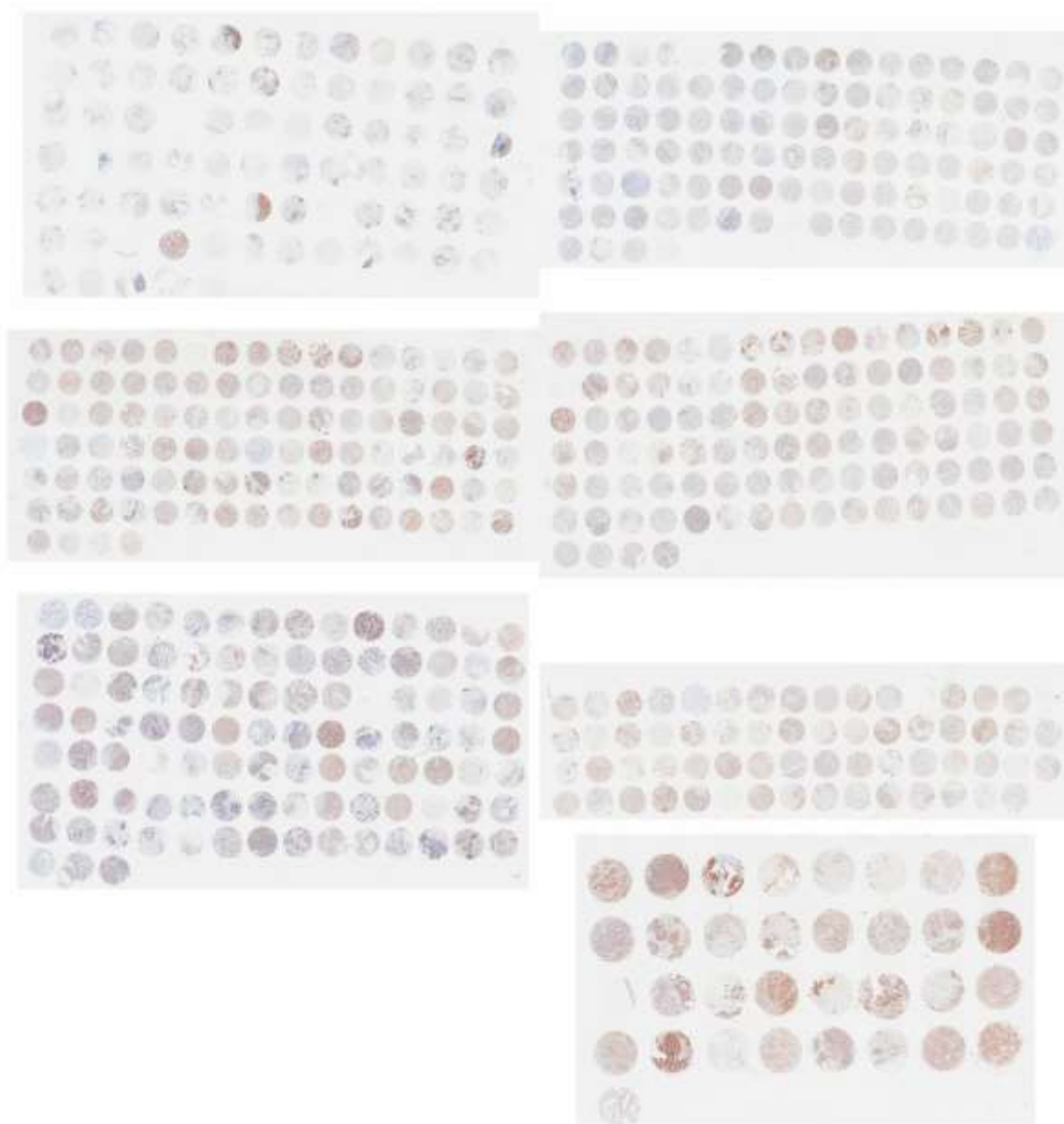

**Figure S1:** The overall tissue micro array result.

Supplement: Supplementary Figure S1 [file BSR-2020-3391_supp.pdf]
